# Skin Acute Wound Healing: A Comprehensive Review

**DOI:** 10.1155/2019/3706315

**Published:** 2019-06-02

**Authors:** Luis Cañedo-Dorantes, Mara Cañedo-Ayala

**Affiliations:** ^1^Research Division, Faculty of Medicine, Universidad Autónoma del Estado de Morelos, Cuernavaca, Morelos, Mexico; ^2^Independent Researcher, Cuernavaca, Morelos, Mexico

## Abstract

Experimental work of the last two decades has revealed the general steps of the wound healing process. This complex network has been organized in three sequential and overlapping steps. The first step of the inflammatory phase is an immediate response to injury; primary sensory neurons sense injury and send danger signals to the brain, to stop bleeding and start inflammation. The following target of the inflammatory phase, led by the peripheral blood mononuclear cells, is to eliminate the pathogens and clean the wound. Once this is completed, the inflammatory phase is resolved and homeostasis is restored. The aim of the proliferative phase, the second phase, is to repair wound damage and begin tissue remodeling. Fibroplasia, reepithelialization, angiogenesis, and peripheral nerve repair are the central actions of this phase. Lastly, the objective of the final phase is to complete tissue remodeling and restore skin integrity. This review provides present day information regarding the status of the participant cells, extracellular matrix, cytokines, chemokines, and growth factors, as well as their interactions with the microenvironment during the wound healing process.

## 1. Introduction

### 1.1. Structure and Function of the Skin

The skin provides a life-protective barrier between the body and the external environment against physical damage, pathogens, fluid loss, and has immune-neuroendocrine functions that contribute to the maintenance of body homeostasis [1]. Its structure is composed of two layers: the epidermis and the dermis. The epidermis contains keratinocytes, melanocytes, dendritic cells, Langerhans cells and other immune cells, sensory axons, and the epidermal-dermal basement membrane [2, 3]. The dermis has the skin appendages, mast cells, fibroblasts, antigen presenting dermal cells, resident and circulating immune cells [4]. Additionally, the dermis includes the extracellular matrix complex that provides support to intercellular connections, cellular movement, and regulates cytokine and growth factors' functions.

Skin innervation consists of a dense network of sensory and autonomic fibers that form tight junctions with keratinocytes and transmit sensations of pain, temperature, pressure, vibration, and itch [5]. Skin circulation is composed of parallel arterial-venous thermoregulatory shunt circulation controlled by tonic adrenergic sympathetic vasoconstrictor and vasodilator nerves that give origin to a subepidermal capillary network that provide oxygen and nutrients to the epidermis and remove CO_2_ and waste products [6]. The lymphatic vessels of the skin consist of lymph capillaries that run horizontally under the epidermis, followed by precollector vessels located deeper in the dermis and lymph collecting vessels in the subcutaneous fat layer. Lymph vessels are connected to the skin local draining lymph nodes, and lymph vessels that exit these lymph nodes converge to the regional sentry lymph nodes before reaching the thoracic duct [7, 8].

## 2. The Healing Process

After injury, skin integrity must be promptly restored in order to maintain its functions. In this process, peripheral blood mononuclear cells, resident skin cells, extracellular matrix, cytokines, chemokines, growth factors, and regulatory molecules participate in the wound healing process. The intricate skin repair process has been organized in three sequential and overlapping steps: the inflammatory phase, the proliferative phase, and the remodelling phase. The inflammatory phase includes cutaneous neurogenic inflammation and hemostasis; these early events start in the first seconds after injury and last approximately 1 hour. Followed by the fast recruitment of neutrophils to the injured tissue during the first 24 hours and its posterior decline during the subsequent week. The progressive infiltration of inflammatory monocytes-macrophages to the wound starts the second day after injury and continues to increase, reaching its maximum during the proliferative phase, starting its decline during the following two weeks, becoming the dominant mononuclear cell in the tissue repair process. Circulating lymphocytes migrate to the skin early after injury reaching a plateau by day 4 and their presence continues for two more weeks before declining. The last phase starts in the second week after injury and includes remodeling the tissue previously formed in the proliferation phase and the organization of a scar in order to restore the skin integrity. This last stage could last for months. This review provides present day information regarding the central role of the resident and peripheral immune cells as well as the microenvironment and their interactions during the wound healing process.

## 3. The Inflammatory Phase (Alarm and Stop the Damage)

### 3.1. Cutaneous Neurogenic Inflammation

**Figure 1:**
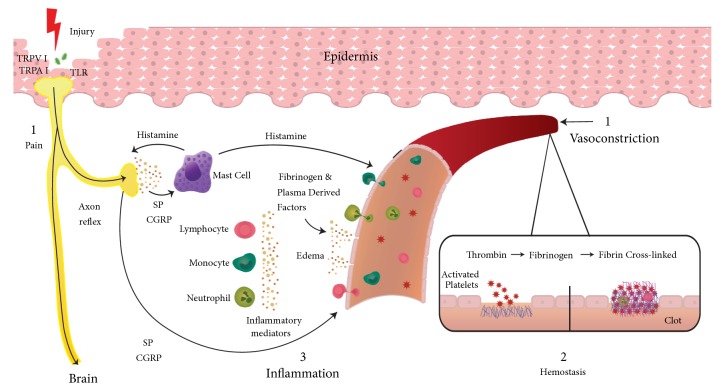
See text.

The* peripheral nervous system* is among the first to respond to a skin injury. Skin cell damage activates transient receptor potential channels TPRV1 and TPRA1 present in primary sensory neuron endings and in other cells such as keratinocytes, mast cells, dendritic cells, and endothelial cells which act as nociceptive receptors [9]. Injury stimulation of sensory neurons generates action potentials that travel orthodromically to the spinal cord initiating* pain*. Action potentials start the axon reflex by traveling antidromically in other axonal branches of sensory nerve endings promoting the release of substance P and calcitonin gene-related peptide from sensory nerve endings [10]. These neuropeptides have three targets: (a) in blood vessels, CGRP act on microvascular smooth muscle fibers promoting vasodilation and increased blood flow, (b) SP causes vascular permeability, edema, and recruitment of inflammatory leukocytes, and (c) SP stimulates mast cells degranulation with discharge of histamine, serotonin, proteases, and other mediators [9, 11–13], promoting increased microvascular permeability of the blood vessels encircling the wound (*redness and warmth*) [14] and facilitating the extravasation of fibrinogen and other plasma derived factors that serve as chemoattractants for the influx of inflammatory cells into the wound (*swelling*) [9, 15, 16]. Additionally, the release of histamine from mast cells triggers the release of substance P and CGRP from sensory nerve endings, implementing the bidirectional link of cutaneous neurogenic inflammation [14] (see Figure 1). The peripheral nervous system continues to have regulatory interactions with mast cells [17], monocyte-macrophages [18, 19], Langerhans cells [20], and lymphocytes [21, 22], as well as microvascular, and other local skin cells during the distinct phases of skin wound healing [23].

### 3.2. Platelets Hemostasis

There are about 160,000-400,000/*μ*l blood platelets, being the second most abundant cells after erythrocytes. An average healthy adult produces 10^11^ platelets per day that circulate around 10 days. Platelets retain many of the RNA metabolic processes of nucleated cells. They contain large amounts of noncoding RNAs, including microRNAs and long noncoding RNAs, and utilize postranscriptional mechanisms to preserve its proteome of approximately 4000 proteins [24]. After they are released into the blood, the progressive degradation of the antiapoptotic protein Bcl-x_L_ determines the lifespan of platelets in the blood, and at the end of their life, they are removed from the circulation in the liver and spleen [25, 26]. Under normal physiological conditions, platelets do not interact with the endothelial surface. Blood constituents tend to migrate toward the center of the blood flow but, given the small size of platelets, they are forced to circulate marginally toward the wall, where the glycocalyx barrier impedes their contact with the endothelial surface [27–29]. Vascular injury exposes the basement membrane proteins and the macromolecules of the extracellular matrix [30]. Platelet membrane surface receptors bind to collagen, activating platelets and producing thrombin that catalyze the initiation of the coagulation cascade [31]. Platelet integrins binding to fibrinogen give origin to fibrin [32, 33] that accumulates with the interstitial collagen, trapping neutrophils, erythrocytes, and other blood components forming the clot [34, 35]. A provisional extracellular matrix is formed by fibrin monomers forming fibrin protofibrils that are stabilized by intermolecular links through the action of Factor XIIIa. In vitro studies suggest that fibrin fibers connect to native collagen type I fibers with cells through *α*_V_*β*_3_ integrins, and this extracellular provisional matrix is used by fibroblasts and endothelial cells to migrate and to promote protomyofibroblast-mediated contraction of the provisional extracellular matrix [36–38]. This initial extracellular matrix is further remodeled by metalloproteinases released from fibroblasts [39] and macrophages, [40] forming a new provisional extracellular matrix to support neutrophil and monocyte migration [41, 42]. Besides hemostasis, degranulation of alpha granules from platelets releases TGF-*β* that acts as an important chemoattractant for the recruitment of various types of immune cells including neutrophils and macrophages [32]. Platelet cell surface receptors participate in cell-cell interaction and microbial recognition and in the release of growth factors such as PDGF, TGF-*β*1, FGF, and VEGF that interact with endothelial cells, neutrophils monocytes, dendritic cells, B and T cells, and natural killer cells, promoting neutrophil activation, pathogen detection, trapping, and modulation of the innate and adaptive immune responses [43, 44].

## 4. The Inflammatory Phase (Eliminate Pathogens and Clean the Wound)

### 4.1. The Role of Peripheral Blood Mononuclear Cells during the Inflammatory Phase

#### 4.1.1. Neutrophils

In healthy human adults, neutrophils constitute 50-70% of all leukocytes. Neutrophils circulate in the blood as quiescent cells with a lifespan of 8-12 hours and 1-2 days in tissues. In the final stages of their lifespan, they are cleared from the circulation in the liver, spleen, and bone marrow [45]. Neutrophils follow platelets as principal effector cells in the initiation of the inflammatory phase at sites of acute inflammation or infection. Their recruitment is initiated by growth factors and chemokines released by activated platelets in the blood clot [46, 47] and by N-formyl peptides released by bacteria and damaged cells [48]. Neutrophil accumulation in the wound increases during the initial inflammatory phase and declines 4 days later [49]. The presence of damage-associated molecular patterns (DAMPs) released during cell damage and necrosis, and the pathogen associated molecular patterns (PAMPs) from bacteria and fungi creates a gradient that is sensed by the numerous neutrophil pattern recognition receptors (PRRs): transmembrane Toll-like receptors, C-type lectins, cytosolic NOD-like receptors, and RIG-like receptors, activating the innate immune response [50]. Neutrophil adhesion receptors (selectins/selectin ligands and integrins) bind neutrophils to the endothelium which then follow the leukocyte recruitment cascade: rolling, adhesion, crawling, and migration to the inflamed tissue [51]. Once in the wound, neutrophils release more neutrophil-chemoattractant mediators to continue neutrophil recruitment [46, 48]. Their concentration reaches more than 5 x10^6^ on the first 24 hours and continues to increase on day 2, making neutrophils the most abundant immune cells present in the wound [49]. In the inflamed tissue, neutrophils capture Fc-receptors of opsonized pathogens facilitating phagocytosis, while reactive oxygen species and antibacterial proteins present in neutrophil granules are released into the phagosome to eliminate the pathogen. In addition to the intracellular killing mechanisms, neutrophils alone or triggered by proinflammatory molecules and platelets, eject neutrophils extracellular traps (NETs), composed of DNA, histones, antimicrobial proteins, and lytic enzymes attached to them. It is through these mechanisms that NETs immobilize and kill microorganisms [45, 52, 53]. Moreover, neutrophils engage in cellular crosstalk via cell-cell contact where numerous cytokines, chemokines, and angiogenic factors activate resident hematopoietic cells, macrophages, dendritic cells, B cells, T cells, and natural killer cells modulating the innate and adaptive immune responses [48].

#### 4.1.2. Monocytes

Circulating human monocytes originate from a monocyte-dendritic progenitor (hMDP) that gives origin to monocytes and a dendritic cell precursor (hCDP) in the bone marrow. Both of these cells are released to the blood and further differentiate in the peripheral tissues as macrophages or dendritic cells [54]. In the blood, three monocyte subsets with different phenotypes have been identified [55]: CD14^++^CD16^−^ classical monocytes (inflammatory) capable of transmigrating and entering tissues, CD14^++^CD16^+^ intermediate monocytes with increased proangiogenic and antigen processing and presentation activities, and CD14^+^CD16^++^ nonclassical monocytes that patrol the vessels with endothelial and tissue monitoring capabilities [56]. These three monocytes differ in size, morphology, and transcriptional profiles [57]. In a recent study of* in vivo* leukocyte kinetics using deuterium labeling, a sequential transition from monocyte progenitors to nonclassical monocytes was reported [58]. This study showed that in the bone marrow, monocyte precursors differentiate into classical monocytes that remained there for a postmitotic maturation phase of 38 hours, which are then released into the blood circulation where they have a short lifespan of 1 day. However, most of these cells leave the blood or die, and only a small proportion of them mature into intermediate monocytes with a lifespan of 4 days. Lastly, most of these cells convert into nonclassical monocytes with a lifespan of 7 days before leaving the circulation or dying [58]. In the steady state, monocyte emigration occurs constitutively, where they can remain within the tissue as monocytes, acquire antigen-presenting capabilities, or mature into macrophages [59, 60]. Circulating monocytes contribute to forming the skin tissue monocyte-macrophage population [61].

#### 4.1.3. Monocyte-Macrophages

After injury, the presence of DAMPs and PAMPs is sensed by tissue-resident macrophages that in turn activate patrolling monocytes to migrate into the wound [62]. Once inside, monocytes release cytokines and chemokines [46, 47] to recruit neutrophils into the wound. The release of neutrophils granule contents promotes the recruitment of inflammatory monocytes that mature into macrophages becoming soon the dominant monocyte-macrophage population in the wound [62–64]. These cells count with great plasticity [65–67], allowing them to differentiate into diverse monocyte-macrophage phenotypes [68] or transdifferentiate to other cell types in response to the particular microenvironments of the wound [64, 69–71]. For instance, macrophages detect PAMPs and DAMPs through their pattern recognition receptors (PRRs) [72], and the production of interferon gamma (IFN*γ*) and tumor necrosis alpha (TNF-*α*) by innate or adaptive immune cells induces macrophages to adopt an inflammatory phenotype (M1) that in turn produces proinflammatory cytokines [65] as well as reactive oxygen and nitrogen species needed to kill and control microbial pathogens [73, 74]. In conjunction with neutrophils, macrophages participate in the removal of bacteria, dead cells, apoptotic neutrophils, tissue debris, and other foreign materials. This* myelomonocytic interaction* is a key component of wound repair [62, 66, 75]. Neutrophils and monocyte-macrophages cooperate as partners in time and space during the initiation, evolution, and resolution of the inflammatory phase [76]. The common progenitor of neutrophils and monocyte-macrophages explains the similar functions they share: phagocytosis, intracellular killing mechanisms, NET formation, similar transcriptional profiles, and cell surface receptors, as well as their participation in the modulation of innate and adaptive immune response [66, 75]. Once the wound is clean, neutrophils collaborate with macrophages to orchestrate the resolution of the inflammatory phase [77]. This stage starts one or two days after neutrophil arrival to the inflamed tissue. Restoring of homeostasis begins with neutrophils releasing microparticles containing proresolving protein annexin A1 and proresolving lipid mediators [78]; apoptotic neutrophils expose phosphatidylserine on the surface designating them for efferocytosis. During this process, neutrophil microparticles transfer their molecules to macrophages upgrading the biosynthesis of proresolving mediators: lipoxins, resolvins, protectins, and maresins that are released into the wound tissue [79, 80]. Efferocytosis of apoptotic neutrophils by inflammatory macrophages stimulates the synthesis of miR-21, promoting the anti-inflammatory phenotype of the postefferocytotic macrophage [81, 82]. This alternative differentiation route creates heterogeneous anti-inflammatory M2 populations [83, 84]. M2a macrophages display an anti-inflammatory phenotype, release IL-10, inhibit the production of IL-1*β* and TNF*-α*, and participate in the resolution of the inflammatory phase. M2b and M2c macrophages mostly contribute to resolving the inflammatory phase by reducing the damage caused by prolonged activation of M1 macrophages [67, 83, 84], and driving the resolution of inflammation. An ordered and well-controlled inflammatory phase is essential for the normal progress of tissue-repair and remodeling phases of wound healing [68, 85].

#### 4.1.4. Lymphocytes

The skin immune system maintains and protects body integrity. Innate immune system, including neutrophils and monocyte-macrophages, provides a non-specific immediate response to pathogens and toxins. Innate cells collaborate with T and B cells of the adaptive immune system that retain specific memory for a long time to fight specifically intracellular and extracellular pathogens.

#### 4.1.5. Innate Lymphocytes

Innate lymphoid cells (ILCs) consist of three family subsets with different cell lineage markers compared to T, B, and natural killer (NK) cells. Group 1 contains NK cells, releases interferon gamma (INF*γ*) and tumor necrosis factor (TNF-*α*), and has cytolytic functions [86, 87]. ILC2 cells are present in healthy skin and increase in number during inflammation. Under IL-33 stimulation, ILC2 responses promote reepithelialization and wound closure [88]. Invariant NKT cells (iNKT) promote skin wound healing by increasing the production of INF-*γ* in the early phase of wound healing, stimulating macrophages and fibroblasts to secrete VEGF and TGF-*β*, increasing collagen deposition, producing myofibroblast differentiation and angiogenesis [89], and preventing neutrophil inflammatory response [90, 91].

#### 4.1.6. CD8+ T Cells

After injury, DAMPs and PAMPs released from damaged cells and pathogens are sensed by a diversity of immune and nonimmune cells present in the skin through a system of pattern recognition receptors that include transmembrane Toll-like receptors and C-type lectin receptors and cytoplasmic proteins, retinoic acid-inducible gene-I-like receptors, and NOD-like receptors (NLRs) [92, 93]. PRRs initiate the immune response through the production of proinflammatory cytokines and antimicrobial peptides, and by recruitment of neutrophils and macrophages [94]. The role of Toll-like receptors in acute skin wounds has been recently reviewed [95]. Early after acute wound, DAMPs and PAMPs antigens are transferred inside the cell by endocytosis and processed by professional and nonprofessional skin resident dendritic cells (DC). Then, professional DC migrate to skin local draining lymph nodes (LN) and present the antigen to naïve CD8+ T cells. Identification of their cognate antigen in the lymph node promotes naïve T cells differentiation into CD8+ skin homing effector memory T cells (T_EM_) and CD8+ central memory T cells (T_CM_). T_EM_ expressing cutaneous lymphocyte antigen (CLA) and CCR4 migrate to the skin wound to mediate pathogen clearance by releasing proinflammatory, immune-regulatory, and microbicidal mediators [96]. Once the antigen sources have been eliminated, the majority of T_EM_ cells die from apoptosis, and a small population of antigen specific T cells expressing CCR8 remains in the skin [97]. Known as CD8+ noncirculating tissue-resident memory T-cells (T_rm_), these cells are the most abundant T-cells present in human skin during resting conditions, estimated as 2 x 10^10^, twice the amount of T cells in the whole blood. Ninety percent of these cells remain in the skin and only 10% of T_rm_ circulate in the blood [98]. In the secondary lymphoid organs T_CM_ express lymph node homing receptors CCR7 and CD62L and proliferate and some differentiate into T_EM_ that migrate to other peripheral lymph nodes providing systemic immunological memory and during local skin inflammation they migrate to the inflamed site [99, 100]. Upon reexposure to the pathogen, local DC present the antigen to skin CD8^+^  T_rm_ that proliferate and recruit T_EM_ from the blood to mediate pathogen clearance [99]. Later, CD8^+^  T_rm_ migrate to the epidermis filling the site previously occupied by delta gamma T cells [101]. T_rm_ are responsible for the first line skin immunological memory defending the skin against reinfection.

#### 4.1.7. CD4+ T Cells

Skin homeostasis and peripheral tolerance to commensals and self-antigens are controlled by skin immunosuppressive CD4+Foxp3+ regulatory T cells (T_regs_) that suppress the abnormal effects of self-reactive immune cells' responses [102, 103]. Circulating T_regs_ expressing the cutaneous lymphocyte antigen (CLA) and the skin-homing receptor CCR6, migrate and accumulate in the hair follicle niche of the skin [104]. T_regs_ increase the expression of epidermal growth factor receptor (EGFR) which favors wound reepithelialization wound closure, modulates tissue inflammation by limiting IFN*γ* production, and reduce the number of inflammatory macrophages [105]. After clearance of the pathogen skin, T_regs_ need IL-7 to remain in the skin and express CD45RO which is indicative of previous antigen exposure, as well as the memory associated markers CD27 and BCL-2; data that characterize them as regulatory resident memory T cells (Treg Trm) in the skin. In adult healthy human skin, approximately 20% of tissue-resident CD4^+^ T-cells are T_regs_  T_rm_ expressing the transcription factor Foxp3, and only 5% of T_regs_ recirculate [104]. In the presence of antigen reexposure, dendritic cells process and present their cognate antigen to resident Treg Trm allowing them to respond rapidly.

CD4+ helper T cells include several subsets: Th1, Th2, Th17, Th22, and Th9 providing host defense by releasing diverse cytokines that in turn promote the release of INF*γ*, defensins, and antimicrobial peptides and supply a protective inflammatory response to protect skin against intracellular and extracellular pathogens [106–108].

#### 4.1.8. B Cells

**Figure 2:**
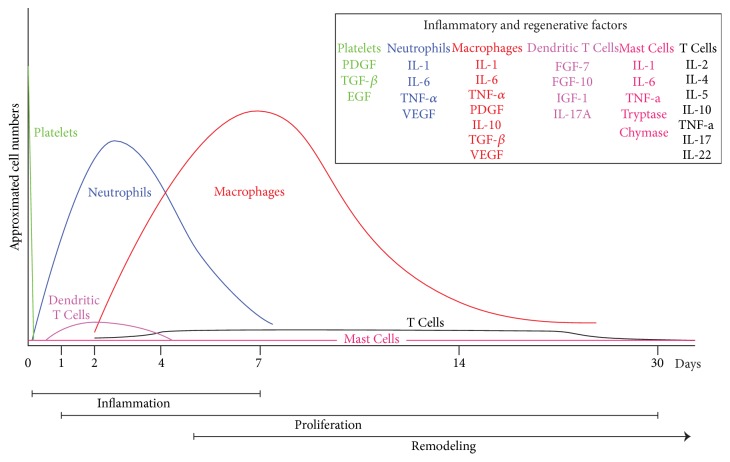
Neutrophil accumulation in the wound increases during the initial inflammatory phase and starts declining 4 days later until the end of the week [49]. Macrophages increase their numbers during the inflammation phase, reach maximum concentration during the proliferation phase, and decline progressively during the remodeling phase, being the most abundant cell in all phases of wound repair [63, 115]. Lymphocytes start increasing their number after injury and reach a plateau at day 4 that continues to be present until the last phase. The skin resident cell number in dendritic cells, mast cells, Trm, and Treg rm is not known, but the presence in the skin of a large population of T memory cells protects the skin against reinfection [99]; further research will be required to clarify their functions in the wound healing process. The approximated time of each of the wound healing phases is illustrated in the bars at the bottom of the graphic [41].

B cells are part of the humoral branch of the immune system. They differentiate into antibody production plasma cells, present antigens to T cells, and regulate local immune responses by releasing growth factors and proinflammatory and anti-inflammatory cytokines [109–111]. In a model of splenectomized wounded mice, it was found that the wound healing process was delayed and the addition of external B cells that produced antibodies against the wounded tissue to these mice, recovers the normal wound repair process [112]. B cells' cytokine production that enhanced the wound healing process has also been reported [113]. An important recent work by Sirbulescu et al [114] demonstrated that B cells are present in the wound bed 4 days after injury persisting up to day 17 after injury. Using a mice model, a 5 mm biopsy was made in the dorsal skin and topical application of mature B cells at the time of injury accelerates the wound healing process by 2-3 days [114] (see Figure 2).

## 5. The Proliferative Phase (Wound Damage Repair)

The proliferative phase is identified by (a) fibroplasia, including fibroblast proliferation and differentiation into myofibroblasts, extracellular matrix deposition, and wound contraction, (b) reepithelialization and epithelial-mesenchymal interaction between keratinocytes and fibroblasts, (c) angiogenesis, including endothelial cell proliferation and new vessel formation, and (d) peripheral nerve repair, consisting in collateral reinnervation and nerve regeneration. Macrophages are the dominant inflammatory cells orchestrating the proliferative phase of skin wound repair [63, 68, 116, 117].

### 5.1. Fibroplasia

Fibroblasts are an ill-defined heterogeneous group of cells with great plasticity and different roles in distinct dermal layers [37, 118]. Fibroblasts are able to respond to tissue soluble extracellular signals such as IL-1, tumor necrosis factor alpha (TNF-a), transforming growth factor beta TGF-*β*1 [119], platelet-derived growth factor (PDGF), epidermal growth factor (EGF), and fibroblast growth factor-2 (FGF-2) released by platelets, macrophage, fibroblast, endothelial cells, and keratinocytes [41, 118–121]. These cytokines and growth factors activate fibroblasts to proliferate and modulate the production of metalloproteinases and inhibitors of metalloproteinases [122]. Mature fibroblasts migrate into the granulation tissue, initiate collagen synthesis, replace the fibrin provisional matrix [41], and differentiate into myofibroblasts increasing collagen deposition and initiating wound contraction [38, 123]. Fibroblasts also sense the strength and direction of mechanical load and translate this information via mechanotransduction signals into gene expression and growth factor production that are expressed as meaningful adaptive responses that transform fibroblast phenotype [37, 124, 125]. For instance, Vimentin, an intermediate filament, activates TGF-*β*–Slug signaling that triggers the epithelial-mesenchymal transition, controls fibroblast proliferation, and increases collagen deposition which in turn activates keratinocyte mesenchymal differentiation and reepithelialization [126].

### 5.2. Reepithelialization

Reepithelialization starts 16-24 hours after injury and continues until the remodeling phase of wound repair [3]. Early after injury, keratinocytes differentiate and migrate between the fibrin clot and the rich collagen dermis while suprabasal keratinocytes located behind the leading edge proliferate to provide more cells to fill the gap. Suprabasal keratinocytes close to the leading edge change shape and migrate on top of basal keratinocytes, becoming leading cells. In the final stages of reepithelialization, cells dedifferentiate into epithelial cells that remain firmly attached to the basal membrane. Cell-cell and cell-ECM interactions, growth factors, and cytokines released by various cell types stimulate keratinocytes to migrate over the provisional matrix deposited in the clot to cover the wound, while keratinocytes at the wound edges begin to proliferate and follow the migrating front [3, 127]. The extracellular matrix plays a key role in the process of reepithelialization [128, 129]. Simultaneously, an active paracrine interaction between keratinocytes, fibroblasts, neutrophils, monocytes-macrophages, and endothelial cells increases the amount of cytokines, growth factors, and other biomolecules to promote the epithelial-mesenchymal interaction between keratinocytes and fibroblasts, where keratinocytes stimulate fibroblasts to release growth factors that in turn stimulate keratinocyte proliferation [118, 122, 130]. Lastly, fibroblasts differentiate into myofibroblasts, increasing collagen deposition and initiating wound contraction [38].

### 5.3. Angiogenesis

During the proliferation phase, the macrophage anti-inflammatory phenotype (M2) emerges as the dominant cellular population, orchestrating the interaction with endothelial cells, fibroblasts, keratinocytes, extracellular matrix (ECM), and peripheral nerves [68, 122, 131, 132]. The reduction of blood supply and the accelerated metabolism of cells working to repair injury cause the wound tissues to become hypoxic, a major stimulus for angiogenesis. Hypoxic conditions stimulate the synthesis of hypoxia inducible factor-1 (HIF1) in macrophages [133, 134], fibroblasts [41, 135], vascular endothelial cells [136], and keratinocytes [137]. The release of proangiogenic factors such as VEGF, VEGFA, FGF2, PDGF, TGF-*β*1, and the metabolic switch of endothelial cells initiate neovascularization [138]. Three endothelial cell types are at the center of angiogenesis: highly migratory tip cells that guide the new growing bud, proliferative stalk cells that elongate the new vessel, and the quiescent falanx cells that form the blood vessel lining [138–140]. Differentiation of endothelial cells into each subtype is primarily guided by the increased presence of VEGF and macrophages [138]. Immature endothelial cells' structures anastomose with other preexisting blood vessels, a fusion facilitated by macrophages [139]. These structures acquire lumens, a new basal membrane, and endothelial cells release PDGF recruiting pericytes, which express receptor *β* (PDGF-R*β*) and cover the new vessels with these mural cells [141], forming new stable blood vessels [138, 142, 143]. Finally, fibroblasts synthesize and deposit new extracellular matrix that gives support to cells and new blood vessels [41, 144], forming the granulation tissue.

### 5.4. Peripheral Nerve Repair

**Figure 3:**
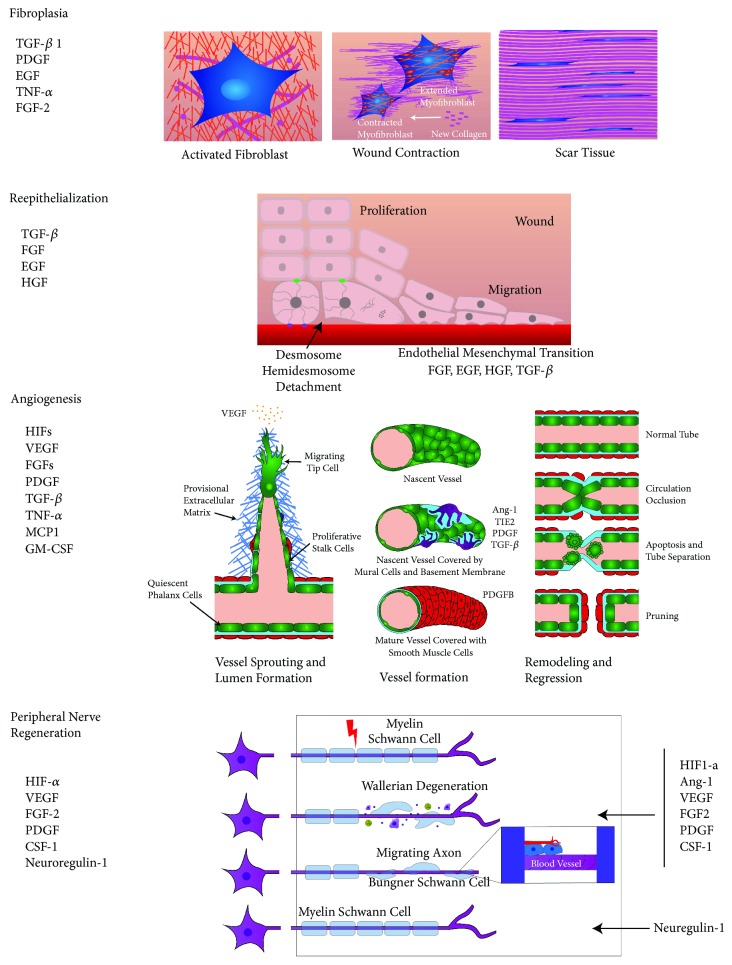
See Sections 5.4.1to 5.4.4.

After injury, severed nerves affect the homeostatic function of the skin. The restoration of neurological functions after traumatic peripheral nerve injury involves two processes: collateral reinnervation and nerve regeneration. Skin denervation stimulates collateral sprouting of nociceptive skin afferents from close undamaged axons to reinnervate the skin [145–147]. In adults, the peripheral nervous system (PNS) is able to regenerate nerve function following an injury, by regrowing the tips of the myelinated two nerve stumps and reconnecting the injured nerve. Monocyte-macrophages, Schwann cells (SC), fibroblasts, inflammatory cytokines, transcription factors, complement, and arachidonic acid metabolites participate in this process [148]. SC store considerable plasticity, and after injury, their myelin sheath is discarded and SC dedifferentiate to a progenitor-like cell to promote axonal regrowth [149]. SC exit the nerve stumps and interact with fibroblasts accumulated at the injury site. Ephrin-B present in fibroblasts contacts the EphB2 receptors of SC and this signaling promotes their directional movement [150]. Simultaneously, SC dedifferentiation induces the release of monocyte chemoattractant protein-1 (MCP- 1), IL-1*α*, IL-1*β*, and pancreatitis-associated protein III (PAP-III) [151] that recruits circulating monocytes/macrophages to the injury site, where these cells release additional factors, thus increasing further monocyte/macrophage recruitment. Macrophages sense the hypoxic environment releasing vascular endothelial growth factor (VEGF) and hypoxic growth factor (HIF) which promote angiogenesis. Subsequently, the SC cords use the new aligned vasculature as scaffold to guide the growing of axons across the bridge between the tips of the two nerve stumps [152–154] (see Figure 3).

#### 5.4.1. Fibroplasia

After acute skin injury, fibrinogen, fibronectin, proteoglycan, and platelets from plasma come into contact with collagen of the extracellular matrix (ECM), forming a fibrin rich early provisional matrix cross-linked with fibronectin (EPM) [41, 155]. Local resting fibroblasts become activated and begin producing collagen that gradually transforms the EPM into a late collagen rich ECM. Activated fibroblasts then deposit collagen and differentiate into myofibroblasts (MFs). Regulated by TGF-*β*1, MFs express alpha smooth muscle actin and muscle myosin, forming intracellular stress fibers that are attached to the fibronexus, a cellular-ECM structure linking intracellular actin filaments to extracellular fibronectin fibrils through transmembrane integrins [38, 41]. MFs bind intracellular stress fibers to extracellular collagen through fibronexus complexes. Additionally, the contraction of stress fibers locally condenses the ECM leaving a space that is replenished with newly synthesized collagen. This process is repeated by other local MFs and the remodeling of small sections of the ECM produces wound contraction [41, 156]. The accumulation of collagen in the wound site leads progressively to an almost avascular and acellular scar formed 80-90% by regularly organized collagen Type I fibers and the rest type III collagen fibers.

#### 5.4.2. Reepithelialization

After injury, basal keratinocytes at the wound edge start the endothelial mesenchymal transition by losing their desmosome connection to each other and the hemidesmosome bond to the basal membrane. The cytoskeleton is then reorganized, losing its cuboidal shape and adopting a flattened morphology with lamellipodia, expressing K6 and K16 that allow them to begin migration into the provisional matrix to fill the gap. Simultaneously, the keratinocytes that remain behind the edge begin to proliferate [127, 128, 157].

#### 5.4.3. Angiogenesis

Wound healing angiogenesis is thought to be an ongoing process in two phases: the proliferation of new blood vessels and the pruning and remodeling phase. Hypoxic conditions after injury stimulate the synthesis of hypoxia inducible factor-1(HIF-1) in vascular endothelial cells, fibroblasts, keratinocytes, and macrophages, followed by the release of angiogenic factors VGEF, FGF, PDGF, and TGF-*β*1 by these cells, triggering neovascularization. Degradation of the vascular basement membrane is followed by pericyte loss and capillary sprouting carried out by three different EC subsets: (1) highly migratory tip cells that guide the new sprout, having VEGF as a major chemoattractant for these cells, (2) highly proliferative stalk cells that elongate the sprout, and (3) the quiescent phalanx cells that form the lining of the blood vessel [138, 139]. Lumen formation within the sprout gives origin to the nascent vessel that, after being covered by pericytes and in conjunction with the endothelial cells, forms a new basal membrane, where a mature vessel is covered by a basement membrane and mural cells [158]. The process of regression and remodeling starts with the contraction of the selected blood vessels. Endothelial cells bind to the same cells of the opposite side of the vessel wall until the lumen is occluded and blood ceases to flow. The EC of the retracting branch disintegrate and EC die from apoptosis, leaving behind a remodeled vascular network [159].

#### 5.4.4. Peripheral Nerve Regeneration

The transection of the peripheral nerve after injury is followed by retraction of the stumps. The poorly vascularized bridge between the stumps becomes hypoxic. Macrophages sense hypoxia and release VEGF, promoting angiogenesis along the original tubes of the bridge formed of basement membrane. Meanwhile, distal stump degenerated by Wallerian degeneration; Schwann cells are detached from the degenerating axons, releasing their myelin, and dedifferentiate into a progenitor-like state. These dedifferentiated SC recruit more macrophages, and together, clean myelin and axon debris. Macrophages promote the vascularization of the bridge between the two stumps, preparing the site for axonal regrowth. Simultaneously, dedifferentiated Schwann cells migrate along the recently formed vasculature, forming the bands of Büngner and guiding the regrowing axons to their original target. Once the axons reinnervate, their original targets, Schwann cells, redifferentiate and remyelinate axons, leading to the termination of the inflammatory response [152, 153].

## 6. The Remodeling Phase (Restoring Skin Integrity)

In this last phase of wound healing, the granulation tissue undergoes a gradual diminishing process. The epidermis, dermal vasculature, nerves, and myofibers of the skeletal muscle are remodeled, forming a functional tissue. Vascular components of fibroblasts and myofibroblast of the granulation tissue are decreased and PBMC cells undergo apoptosis or leave the wound. Similarly, the amounts of proteoglycans and glycosaminoglycans that provided structural and hydration role are diminished. Collagen metalloproteinases released by fibroblasts and macrophages degrade collagen Type III of the granulation tissue and replace it with collagen Type I, which is further reorganized into paralleled fibrils, forming a low cellularity scar [42, 68, 117, 160]. This last phase can last for months (see Figure 3).

### 6.1. Common Complications of Normal Skin Acute Wound Healing Process

Two common complications are associated with alterations of the normal skin acute wound healing process: fibrosis and chronic skin wounds. These alterations affect millions of people around the world, representing a major health challenge and healthcare expenditure for patients and countries globally. Some challenging problems will be briefly addressed in the following paragraph.

Fibrosis is characterized by excessive production of extracellular matrix. In human skin, fibrosis is recognized as hypertrophic scars and keloids. Hypertrophic scars grow after surgery, trauma, or burns causing deformity and contractures across the joints. Keloids develop as profuse scarring that extends beyond the limits of the original injury causing deformity, pruritus, and hyperesthesia [161]. In the setting of normal skin wound healing, tissue remodeling, scar formation, peripheral blood mononuclear cells, resident skin cells, extracellular matrix components, and signaling pathways are orchestrated in a highly regulated process to restore tissue homeostasis. In contrast, skin fibrosis presents a deregulation of this process, including: (1) pathologically sustained inflammation due to the permanence of inflammatory macrophages and altered communication between macrophages, fibroblasts, and epithelial and endothelial cells [68]; (2) increased fibrosis with the constant presence of activated myofibroblasts and hyperproduction of collagen [38, 68, 162]; (3) altered signaling pathways of fibroblast growth factor (FGF), hepatocyte growth factor (HGF), epidermal growth factor (EGF), and transforming growth factor-beta (TGF-*β*); (4) persistent epithelial-mesenchymal transition (EMT) [38]; (5) altered extensive communication between the different ECM components, growth factors, and the cells immersed in it; and (6) Changed composition of the ECM, altering the mechanotransduction mechanisms between ECM and cells [163–165]. A recent publication summarizes the main factors involved in fibrosis: macrophages, myofibroblasts, matrix, mechanics, and miscommunication [166].

The second complication is chronic nonhealing wounds, clinically known as venous and arterial leg ulcers, pressure sores, and diabetic foot ulcers. We will refer briefly to the most important pathophysiological challenges of diabetic foot ulcers.

Diabetic foot ulcer is a serious and expensive complication of diabetes associated with peripheral vascular disease and neuropathy in the lower limbs that frequently end in amputation [167]. Diabetes hyperglycemia disrupts the homeostasis of glucose metabolism in endothelial cells, neurons, Schwann cells, and peripheral blood mononuclear cells (PBMC) because these cells are not able to reduce the intracellular glucose transport in the presence of hyperglycemia [168]. The excess of glucose interrupts the normal flux of glycolysis. This causes intermediates to be diverted into collateral pathways that increase the production of reactive oxygen species, peroxynitrite, and toxic advanced glycation end products (AGES). The increased activity of PKC causes vascular abnormalities and proinflammatory gene expression [169]. These toxic alterations lead to vascular, nerve, and PBMC damage, manifested as vascular disease, neuropathy, and immune alterations. Hyperglycemia induced changes are also manifested in the skin [170], keratinocytes [171], and fibroblasts [172]. Thus, diabetic foot ulcers exhibit a chronic inflammatory status and altered molecular environment including growth factors, cytokines, and proteases [173] and inflammatory cells present a dysfunctional phenotype [174, 175].

## 7. Conclusion and Perspectives

Experimental work of the last two decades has revealed the general steps of the wound healing process. All cells, tissues, cytokines, chemokines, and growth factors of the skin participate in the wound healing process, revealing redundant and pleiotropic functions and interactions in many of the cellular and extracellular participants in wound repair. Further understanding of this complex network will elucidate how skin cell interaction with the changing tissue microenvironment defines their phenotype in every stage of tissue repair. Present knowledge has revealed that when cells are healthy, the inflammatory phase is well orchestrated, lasting only a few days, and the following stages of tissue repair: reepithelialization of the wound, granulation tissue formation, wound contraction, and scar formation, proceed normally. However, when cells are dysfunctional, as in diabetes, the inflammatory process is extended, the integrity of the skin is not restored, and ulcer or pathological fibrosis occurs. Macrophages are the dominant cells present in all phases of tissue repair. They have an essential regulatory role and are therefore seen as important therapeutic targets to control the wound healing process in the future.

## Abbreviations

ANG-1: Angiopoietin 1
TIE-2: Angiopoietin receptor
CSF-1: Macrophage colony stimulating factor-1
EGF: Epidermal growth factor
FGF-2: Fibroblast growth factor-2
FGF-7: Fibroblast growth factor-7
FGF-10: Fibroblast growth factor-10
GM-CSF: Granulocyte-macrophage colony-stimulating factor
HIF: Hypoxia inducible factor
HGF: Hepatocyte growth factor
IL-1: Interleukin-1
IL-2: Interleukin-2
IL-4: Interleukin-4
IL-5: Interleukin-5
IL-6: Interleukin-6
IL-10: Interleukin-10
IL-17: Interleukin-17
IL-22: Interleukin-22
ILF: Insulin-like growth factor-1
MCP-1: Monocyte chemoattractant protein
PDGF: Platelet derived growth factor
PDGFB: Platelet derived growth factor subunit B
TGF-*β*: Transforming growth factor beta
TNF-*α*: Tumor necrosis factor alpha
VEGF: Vascular endothelial growth factor.
